# A Novel and Reliable Pixel Response Correction Method (DAC-Shifting) for Spectral Photon-Counting CT Imaging

**DOI:** 10.3390/tomography10070089

**Published:** 2024-07-22

**Authors:** Navrit Johan Singh Bal, Imaiyan Chitra Ragupathy, Trine Tramm, Jasper Nijkamp

**Affiliations:** 1 Department of Clinical Medicine, Aarhus University, 8200 Aarhus, Denmark; nbal@clin.au.dk (N.J.S.B.); imaiyancr@clin.au.dk (I.C.R.); tramm@clin.au.dk (T.T.); 2 Danish Centre for Particle Therapy, Aarhus University Hospital, 8200 Aarhus, Denmark; 3 Department of Pathology, Aarhus University Hospital, 8200 Aarhus, Denmark

**Keywords:** spectral computed tomography, K-edge imaging, photon-counting detector, cone-beam computed tomography, pixel response correction, Medipix3

## Abstract

Spectral photon-counting cone-beam computed tomography (CT) imaging is challenged by individual pixel response behaviours, which lead to noisy projection images and subsequent image artefacts like rings. Existing methods to correct for this either use calibration measurements, like signal-to-thickness calibration (STC), or perform a post-processing ring artefact correction of sinogram data or scan reconstructions without taking the pixel response explicitly into account. Here, we present a novel post-processing method (digital-to-analogue converter (DAC)-shifting) which explicitly measures the current pixel response using flat-field images and subsequently corrects the projection data. The DAC-shifting method was evaluated using a repeat series of the spectral photon-counting imaging (Medipix3) of a phantom with different density inserts and iodine K-edge imaging. The method was also compared against polymethyl methacrylate (PMMA)-based STC. The DAC-shifting method was shown to be effective in correcting individual pixel responses and was robust against detector instability; it led to a 47.4% average reduction in CT-number variation in homogeneous materials, with a range of 40.7–55.6%. On the contrary, the STC correction showed varying results; a 13.7% average reduction in CT-number variation, ranging from a 43.7% increase to a 45.5% reduction. In K-edge imaging, DAC-shifting provides a sharper attenuation peak and more uniform CT values, which are expected to benefit iodine concentration quantifications.

## 1. Introduction

In the past decade, computed tomography (CT) research has been focused on replacing energy-integrating detectors (EIDs) with energy-discriminating photon-counting detectors (PCDs). With PCD-CT imaging, you can reach a higher spatial resolution, signal-to-noise ratio, and contrast-to-noise ratio compared to EID-CT [1]. Furthermore, its energy discriminating ability allows you to acquire several energy bins during a scan, which allows for material decompositions, for example, of fat, water, and lipid concentrations [2], or to distinguish between multiple contrast agents [3].

PCDs typically consist of multiple chips which each have a two-dimensional readout grid (an application-specific integrated circuit, ASIC) coupled to a semiconductor such as cadmium telluride (CdTe) or silicon (Si), which generates an electrical charge. The energy discrimination is implemented by thresholding the electrical charges using two or more counters. For normal operation, the first counter is set above the electrical noise of the system. Subsequent counters can be set to energy specific values, making sure that the electrical charge created by a photon is counted only when it exceeds the threshold value. One example of a PCD chip is the Medipix3RX, which consists of 256 × 256 pixels, with a pixel pitch of 55 μm and two threshold counters per pixel [4]. In spectroscopic configurations, 2 × 2 pixels are combined, resulting in a 110 μm pixel pitch and eight counters per pixel [5]. The chips can be tiled together to create a large field of view (FOV). The clinical devices using this technology are the MARS Bioimaging system [6] and the spiral breast CT imaging system [7].

The thresholds for the counters are set per chip and are defined as integer numbers (typical range 0–255 or 511), which are also known as digital-to-analogue-converter (DAC) units. The relationship between a DAC and actual keV threshold is close to linear and needs to be calibrated for each chip. Several methods of calibration exist, of which the following three are most common: (1) kVp scanning with a polychromatic X-ray source, where you change the DAC from high to low, searching for the setting where a pixel starts to count photons, which corresponds to the kVp [8]; (2) the X-ray fluorescence (XRF) of pure metal foils, where you search for the DAC corresponding to the characteristic K-shell electron transition energies of the metals [8]; (3) the use of monochromatic/radioactive X-ray sources, where the highest DAC with substantial counts corresponds to the source energy. With two or ideally more points within the energy range of interest, their linear relation can be determined.

While DAC settings are set per chip, their actual response varies per pixel. This means that, on average, the image acquired with a certain threshold corresponds to a specific keV value, but the image will be noisy because individual pixels represent a specific range of keV threshold values. These pixel response variations result in ring artefacts in the reconstructed images; therefore, projection images need to be corrected first. Basic flat-field correction (FFC) is not sufficient because the count response of the pixels is energy- and flux-dependent and because of the beam hardening that is present in the projection images.

One well-known method to correct for the individual pixel responses is the so-called signal-to-equivalent thickness calibration method (STC) [9,10,11]. In STC, a set of homogeneous flat absorbers (for example, sheets of aluminium or PMMA) are imaged with a PCD, with a fixed set of thresholds and X-ray tube settings. The non-linear relationship between absorber thickness and photon counts is subsequently fitted per pixel. To apply the STC method to imaging data from samples, you replace the counts in the projection images with the corresponding thicknesses. This method has an advantage in that it corrects both for pixel response and for beam hardening. As the STC method is based on calibration data, it is highly dependent on the stability of the imaging system, including the X-ray source and the detector response.

Alternative methods focus on the removal of ring artefacts either by correcting the sinogram data or the reconstructed image [12]. Corrections of the sinogram data have particularly been shown to do a respectable job removing ring artefacts, but challenges can be present at high-contrast edges and with small objects or particles within the scanned object.

The above-described methods are mostly focused on correcting data which are non-spectral, like EID scans, or on correcting individual spectral threshold/bin data from PCDs. These correction methods do not explicitly incorporate individual pixel spectral responses, which are the cause of the problem in the first place. When PCDs are used to acquire multi-spectral data, with more than two thresholds there is a risk that individual threshold correction can lead to the corruption of the spectral integrity of the data.

In this work, we introduce an image post-processing method that does not require predefined calibration images. Instead, we used flat-field images, acquired just before or after the scan acquisition of a sample, to determine the individual pixel response in terms of DAC-to-keV at the time of scanning. In the next step, the spectral projection data were fitted per pixel and per projection using cubic B-splines, and the counts for each pixel were corrected per keV threshold using the individual pixel response. With this method, we assumed that the X-ray source and detector response were stable within the scan acquisition, while we mitigated fluctuations over longer time periods. This method allows for freedom in choosing the X-ray source settings and the number and level of thresholds.

The purpose of this study was to describe the method, illustrate its performance, and compare the resulting quality of the reconstruction images when using standard flat-field correction and PMMA-based STC. For this purpose, we used a custom phantom with different plastics, water, and oil and performed the K-edge imaging of a phantom containing different concentrations of iodine.

## 2. Materials and Methods

### 2.1. Experimental Setup

**Figure 1:**
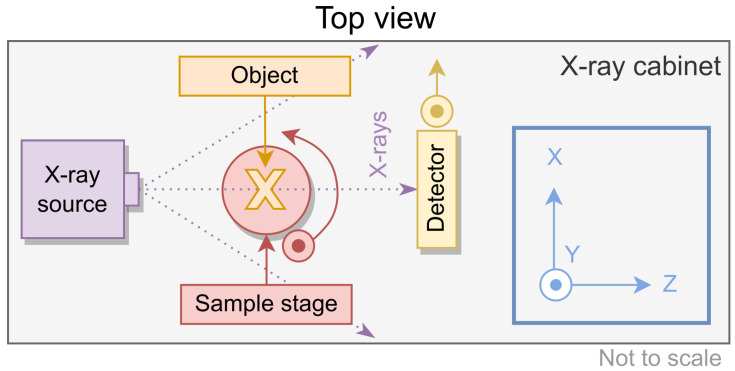
Schematic overview of the experimental setup from above, with the coordinate system shown on the right.

A schematic view of the experimental setup is shown in Figure 1. A radiation-shielded X-ray cabinet (EZ-Access, Metrix NDT Ltd., Loughborough, UK) was used, equipped with a static microfocus X-ray source with a focal spot of 5–7 μm (L9421-02, Hamamatsu Photonics K.K, Hamamatsu, Japan), a sample stage with four degrees of freedom (X, Y, Z, R), and a detector stage with three degrees of freedom (X, Y, Z). A 500 μm thick aluminium filter was used for all measurements to reduce the proportion of low-energy photons.

For imaging, we used two Medipix3 detectors from two different manufacturers. The first was an air-cooled 2 × 2 grid layout detector with a 500 μm silicon (Si) sensor biased to +150 V (Amsterdam Scientific Instruments B. V., Amsterdam, the Netherlands). The second detector had a 2 × 5 layout, was water-cooled, also had a 500 μm Si sensor, and its bias voltage was +150 V (WidePIX, Advacam S.R.O., Prague, Czech Republic). Both detectors had a pixel pitch of 55 μm, with chips of 256 × 256 and two energy thresholds. Each chip in each detector was first calibrated using XRF measurements that were made using pure metal foils of Cu (8.0 keV), Mo (17.4 keV), Rh (20.1 keV), and Sn (25.1 keV) [8].

The imaging of samples was performed using step-and-shoot, meaning that the sample stage was static during the acquisition of projection data. To acquire spectral data with only two thresholds per chip, we acquired multiple projection images at each rotation angle of the sample, where the thresholds in keV were changed for each projection. Before and after the acquisition of projection data, a set of ten open images with the same threshold settings was acquired.

### 2.2. Image Post-Processing (DAC-Shifting)

**Figure 2:**
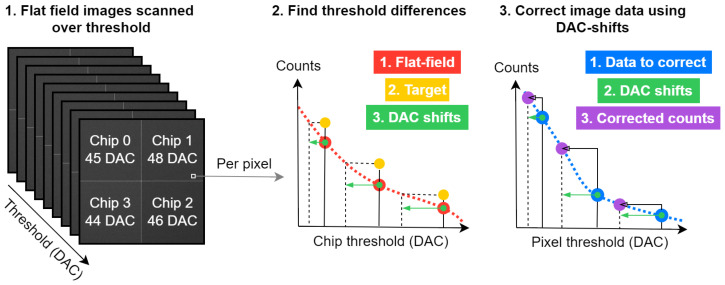
This shows the process of DAC-shifting. The input includes energy-dependent flat-field data and energy-dependent image data. The output is a set of corrected images. The red points represent the flat-field data of a single pixel and threshold. A B-spline with a second-order polynomial is fitted to these points. The target is the median counts as a function of energy for the entire detector per threshold. The DAC shifts are the per-pixel differences in threshold between the flat-field images and the target. These DAC shifts are used to find the corresponding point in the spline fit of the input data. This point is the corrected value in counts. This process is repeated for all pixels and thresholds to correct the images.

Our proposed image post-processing method, called DAC-shifting, consists of two major steps, see Figure 2. In step 1, the individual pixel responses were modelled using flat-field data. Here, we assumed an ideal setup, where the X-ray spectrum and flux are homogeneous over the entire detector. We also assumed that the XRF chip calibration assures that image acquisition with a certain threshold in keV results in comparable photon counts between the chips. With these assumptions, the ideal flat-field image is a homogeneous image with a certain number of photon counts for each threshold. For each specific threshold, the median number of photon counts over the entire image was taken as the ideal target counts per threshold. Subsequently, the individual pixel responses were modelled using cubic b-spline fits through the different flat-field thresholds, using the chip DAC units on the x-axis and the pixel specific counts on the y-axis. The individual pixel fits were then used to find which DAC setting per threshold corresponds to the ideal target count. The primary outcome of this step was a look-up table (LUT) that describes, for every individual pixel, which DAC unit corresponds to which keV threshold.

In step 2, the projection data were processed and corrected. For each projection angle, the individual pixel’s data were fitted using a cubic b-spline, again using the chips’ DAC units for different thresholds on the x-axis and pixel-specific counts on the y-axis. With the LUT from step 1, the counts in each pixel and threshold were then corrected such that every pixel reflected the same keV threshold. As a result, all the projection data were corrected using the individual spectral responses of the pixels. For the FFC during image reconstruction, the ideal median counts per threshold were used instead of the actual flat fields.

It is important to note that the DAC-shifting method was performed separately for each counter. This was necessary because the individual pixel responses were also counter-specific.

### 2.3. Phantom Imaging

To match the size of the detector, a small custom phantom was produced. It was a 20.0 mm diameter cylinder with a lid, with both parts made of PMMA-c. It had seven 4.0 mm diameter holes along the length of the cylinder, which held other materials. These materials were water, sunflower oil, PC, PEEK, POM-c, and PTFE, and, centrally, air and were chosen due to their relatively similar X-ray attenuation coefficients and densities.

The phantom was imaged using both detectors, with a distance source detector (DSD) of 171 mm and distance source object (DSO) of 123 mm. In total, 720 projection angles (0.5-degree spacing) were acquired for each of the five acquisition angles using both threshold counters, resulting in ten threshold projections per angle in total. The exposure time per threshold was optimised to achieve similar counts for the different thresholds (Table 1).

The X-ray source was set to 50 kVp and 160 μA. In each imaging session, the phantom was scanned twice, first using a static detector position, and then followed by an acquisition with random detector shifts between −5 and +5 pixels in the x- and y-direction, similar to [13]. These random detector shifts were used as a hardware solution to mitigate ring artefacts, which comes at the cost of more noise in the reconstructed image. For a comparison between both PCDs, only data from four of the ten chips in the WidePIX detector were used. For one detector, these measurements were performed three times within a time span of 25 days. This was done to showcase the stability of the system. An overview of the measurements taken can be found in Table 2.

**Figure 3:**
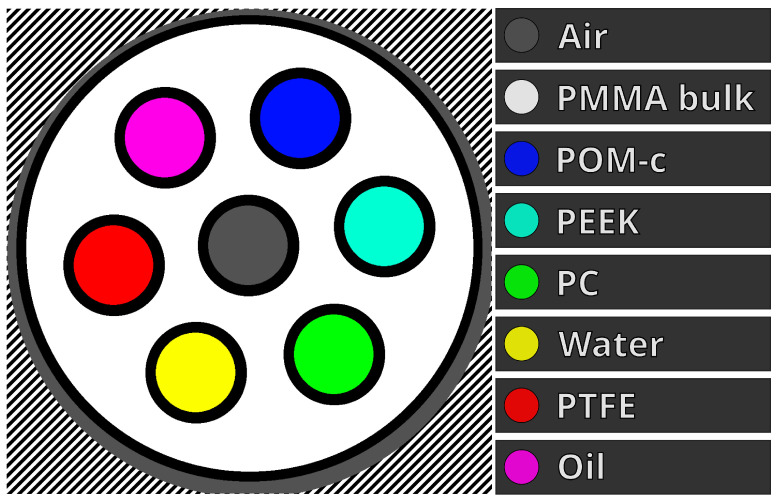
Labelled diagram of a reconstructed slice of our custom microCT phantom. Each material has one unique colour; the excluded regions are the black margins. The black–white diagonal lines show the non-valid reconstruction region.

For image analysis, materials were segmented out using combinations of circular masks defined by centres and radii, see Figure 3. A margin (black border) was used around each selection because the materials were not perfect cylinders due to the manufacturing method used and the cylinders were not perfectly aligned with the z-axis.

**Figure 4:**
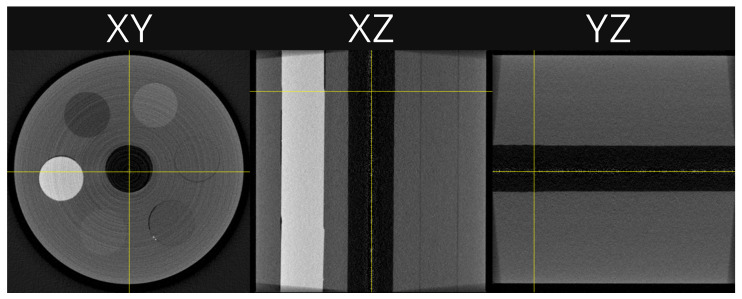
An example reconstruction showing slices in each plane, XY, XZ, and YZ. The grey scale is set to the full data range. The horizontal yellow line in the XZ plane shows the slice used in subsequent plots.

For the sake of illustration, Figure 4 shows an example of a CT reconstruction in all three spatial planes. The example reconstructed slice shown throughout this work was fixed on the same XY plane. This was chosen as it is a good representation of an average slice. It also contains two distinct relatively high attenuation dots that are a few pixels in size, in the lower right quadrant, on the edge of the PC (polycarbonate). These can be used to visually assess the reconstructed image’s quality. The viewing window is fixed at −1000–1800 HU for all slice plots, unless otherwise stated.

### 2.4. Image Reconstruction

For image reconstruction, we used the TIGRE toolbox [14]. Scans were reconstructed per threshold, meaning that each reconstruction represented the counts above a certain energy threshold, not an energy bin. For reconstruction, we used the standard FDK (Feldkamp, Davis and Kress) option with a cosine filter. We also employed iterative FISTA (fast iterative shrinkage thresholding algorithm) reconstruction using the FDK as the prior image, 100 reconstruction iterations, a hyper parameter of $2\times 10^{-4}$, and 75 denoise iterations (‘tviter’) in every reconstruction iteration. For other parameters, the defaults were used. The chosen parameters were empirically optimised to balance the trade-off between image sharpness and noise. All reconstructions were $512^{3}$, with isotropic voxels of 42 μm.

We applied five different post-processing methods to the projection data before reconstruction. The first was standard FFC. The second method was a detector-based STC conversion of the counts to equivalent thicknesses of PMMA, STC-D. For the STC calibration we acquired five projection images per threshold using the following thicknesses of PMMA: 0, 0.8, 1.6, 3.0, 6.0, 9.0, 12.0, 15.0, 18.0, 21.0, 24.0, 27.0, and 30.0 mm. The median number of photon counts over the five projections for each thickness was divided by the median counts measured at thickness 0. We subsequently modelled individual pixel responses using the global hyperbolic interpolation method from [15]. For STC-D, one STC calibration curve was fitted for every threshold using the medians of the detector. This reconstruction step corrected for beam hardening but not pixel response variation. The third method was individual pixel STC conversions, where calibration curves were fitted for each pixel separately, STC-P. This reconstruction step was aimed at correcting both for beam hardening and pixel response. As the fourth, the DAC-shifting method was applied to correct for the pixel response only (DAC-shifting). In a last step, the STC-D and DAC-shifting were combined to correct for beam hardening and pixel responses (DAC and STC). With five post-processing options and two reconstruction methods, each projection set was reconstructed ten times.

The imaging data of the phantom were converted to Hounsfield units (HUs). HU calibration for EIDs is relatively straightforward, but this is not the case for spectral data. For PCDs with configurable energy thresholds, the HUs can change with respect to the energy threshold. We chose to use the median water and air values for each energy threshold as calibration reference points. The calibration becomes

(1) $$HU\left(E\right)=1000\times \frac{\mu \left(E\right)-\mu_{water}\left(E\right)}{\mu_{water}\left(E\right)-\mu_{air}\left(E\right)}$$

where μ(E) are linear attenuation coefficients and *E* is the energy the threshold of the detector is set to. With this approach, the median water and air *HU* values for each threshold are fixed to 0 and −1000, respectively. This was carried out for every reconstruction independently.

### 2.5. Statistics

In this study, we compared the effect of five different options to improve image quality: reconstruction type, DAC-shifting, STC-D, STC-P, and detector motion. For a presentation of the images’ quality and the spectral differences between the materials, the median HUs over threshold were plotted, together with the 95th percentile range of the HU values present in the different materials. For this step, cylindrical regions of interest were used; see Figure 3. The first image quality metric was the range of values for a single material within a reconstruction. We arbitrarily chose the 95th percentile range in HUs and calculated the absolute difference between the upper and lower value as the percentile size. The percentile size quantifies the precision of the system; the closer to zero it is, the better. To represent the spectral information of each material, we took the median across the different thresholds for each material within a reconstruction. To represent the overall image quality of a reconstruction, the mean percentile size across the different materials was used.

To rank the impact of each option, we evaluated their effectiveness in improving the image quality. To do so, we calculated and compared the mean relative change in percentile sizes of the reconstructions, along with the interquartile ranges between each pair, without and with each parameter. These data will be named the ‘effectiveness score’ in this paper and will be shown in percentages. For example, all FDK reconstructions versus all FISTA reconstructions. Negative values show a reduction (improvement) in percentile sizes.

In addition to this, we calculated *p*-values using a *t*-test on two related samples (without and with each parameter) to quantify the similarity of the pairs to each other. ‘This is a test for the null hypothesis that two related or repeated samples have identical average (expected) values’ [16]. A *p*-value below 0.01 was deemed significant.

### 2.6. Spectral K-Edge Experiment with an Iodine Solution

This part aimed to illustrate the capability of the DAC-shifting method to enhance the spectral profile (the attenuation coefficient (cm^−1^) as a function of energy (keV)) of the iodine K-edge (33.2 keV) in both 2D and 3D spectral CT imaging.

The first experiment utilised 2D spectral imaging to examine a 3D-printed cuboid vessel made of polylactic acid (PLA), with a uniform cavity thickness of 6.5 mm, containing the iodine solution at a concentration of 270 mg iodine/mL in water (Visipaque ™, Iodixanol, GE Healthcare, Chicago, IL, USA). This setup allowed for consistent path length measurements of the iodine solution for each pixel of the detector, employing 2×2 chips. Imaging was performed using an X-ray source set at 90 kVp and 89 μA, with energy thresholds ranging from 8.0 to 56.0 keV in 0.5 keV increments, each with a total exposure duration of 48 s. A flat-field correction was applied by scanning the same cuboid vessel in an empty state under identical parameters, enabling an isolated assessment of the iodine solution’s attenuation. Additionally, flat-field images were used to construct a LUT, which is essential for the initial DAC-shifting step. The outcome of this experiment focused on the effect of DAC-shifting on the spectral profile of the entire detector matrix, each discrete chip, and individual pixels.

In the subsequent experiment, a 3D-printed (polylactic acid) cylindrical phantom with a 20.0 mm diameter was utilised to perform 3D spectral CT imaging. This phantom, with nine cylindrical cavities, each 4.0 mm in diameter, was filled with varying iodine concentrations (135.0, 67.50, 33.75, 16.88, and 8.45 mg/mL), water, and three empty cavities. Guided by the spectral profile of the iodine solution established in the 2D spectral imaging experiment, thresholds were selected for 3D spectral CT acquisition, as presented in Table 3, specifically targeting the iodine K-edge.

The phantom was imaged using the following system configuration, with detector motion and using the step-and-shoot acquisition mode:

- Tube voltage = 90 kVp;
- Tube current = 89 μA;
- DSO (distance source object distance) = 123.0 mm;
- DSD (distance source detector) = 163.0 mm;
- Projections = 720;
- Rotation angle = 360°
- Reconstructed voxel size = 0.0415 mm^3^.

**Figure 5:**
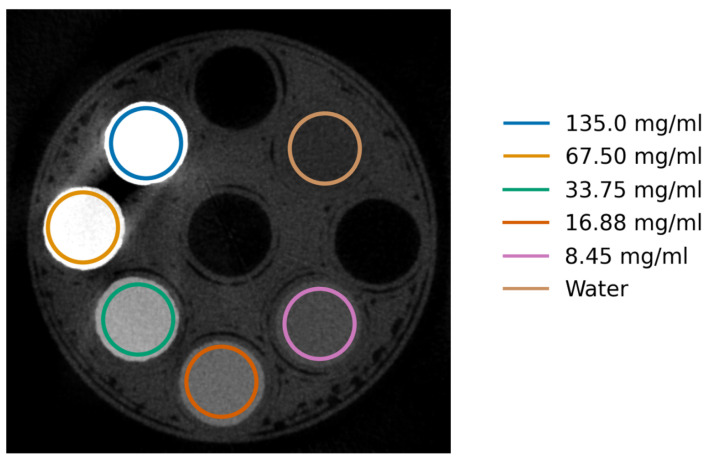
DAC-shifted FISTA-reconstructed CT slice of the iodine phantom at 33.0 keV, with the manually segmented regions marked in circles. Its concentrations are ordered anti-clockwise starting from 135.0 mg/mL.

Ten flat-field images were acquired before and after the projection images. CT reconstructions were then carried out as described in Section 2.4, using both reconstruction algorithms without and with DAC-shifting. Cylindrical regions of interest (volume = 79 mm^3^), as shown in Figure 5, were used to evaluate each iodine concentration.

The quantification of different iodine concentrations was achieved by examining their Hounsfield units at 33.0 keV, the energy level closest to the K-edge peak. A linear regression model (y=mx) was used to evaluate the correlation between iodine concentrations and CT numbers. The y-intercept was set to the HU of water (zero). Further, the R^2^ values without and with DAC-shifting methods at 33.0 keV were compared using Fisher’s r-to-z transformation, calculating their z-score and corresponding *p*-value to determine the significance of the difference between the two correlation coefficients.

## 3. Results

### 3.1. Out-of-the-Box Imaging

**Figure 6:**
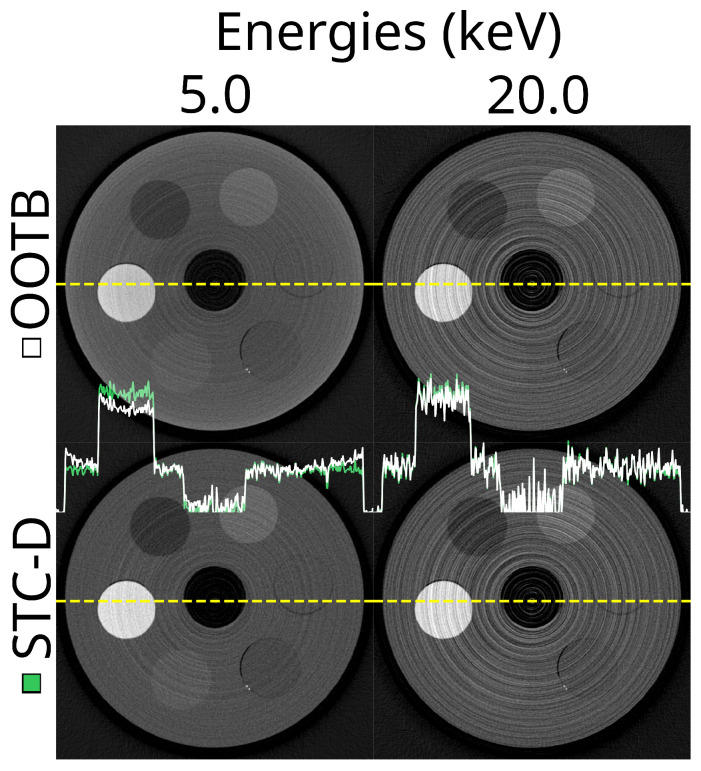
These are the results of applying a beam hardening correction method (STC-D) to CT reconstructions. The top row shows reconstructed slices at 5.0 keV and 20.0 keV without any beam hardening correction (out-of-the-box, OOTB). The bottom row shows the same reconstructions after applying the STC-D beam hardening correction. In the centre of the image are profile plots. These are the profiles of the uncorrected (white, OOTB) and corrected (green, STC-D) reconstructions along the yellow dotted lines. The detector was not moved; no DAC-shifting or any form of STC was used, while FDK was used for reconstruction. This uses the ASI detector (‘ASI-1’ data). The beam hardening correction (STC-D) visibly reduces artefacts in the reconstructed images compared to the out-of-the-box (OOTB) results.

To set the reference, we started with a CT reconstruction showing the out-of-the-box (OOTB) experience (upper row of Figure 6). Apart from manufacturer-specific procedures, the only calibration carried out at this stage was a basic energy calibration per chip using XRF. This only ensured that the chips were aligned in terms of energy to the first order.

**Figure 7:**
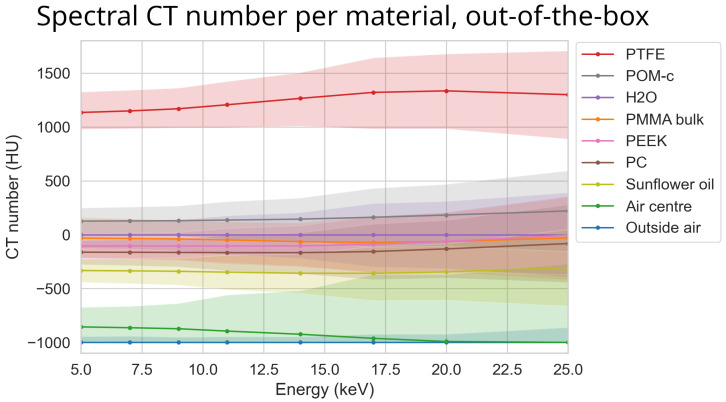
The median reconstructed CT number per material over different energies using the ASI detector for the most basic out-of-the-box configuration. The colour bands represent the 95th percentile range of each material. The scan (‘ASI-1’ dataset) was made using FDK reconstruction, no detector motion, no signal-to-thickness calibration, and no DAC-shifting. The corresponding median of all percentile sizes for these data is 234 HU.

The spectral profile of the different materials can be seen in Figure 7. Note that, despite the increasing exposure time used for increasing thresholds, there was a substantial increase in percentile size with increasing thresholds. Also note that the 95th percentile bands overlap each other for most materials.

### 3.2. Effectiveness Scores

In Table 4, an overview of the effectiveness scores of the investigated options on the different datasets can be seen. In the following sections each option is separately illustrated.

### 3.3. Stc-D Beam Hardening Correction and Detector Motion

The effect of the STC-D beam hardening correction is visualised in Figure 6. From the profile lines in the figure, especially at the 5 keV threshold, there was clearly beam hardening present in the OOTB reconstruction, which was corrected using STC-D. The overall effect of STC-D on the effectiveness score was limited (Table 4), as the beam hardening was present mainly in the PMMA bulk and the PTFE, but not in the other materials.

**Figure 8:**
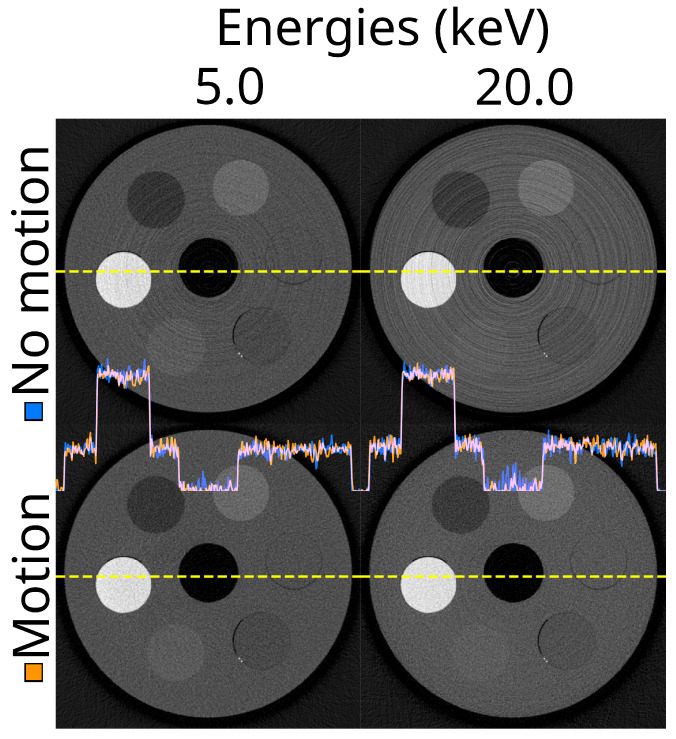
The same reconstructed slice for two energy thresholds (5.0 and 20.0 keV), without and with detector motion. The reconstructions are from the ASI-1 data, with STC-D beam hardening correction, DAC-shifting, and FDK reconstruction.

**Figure 9:**
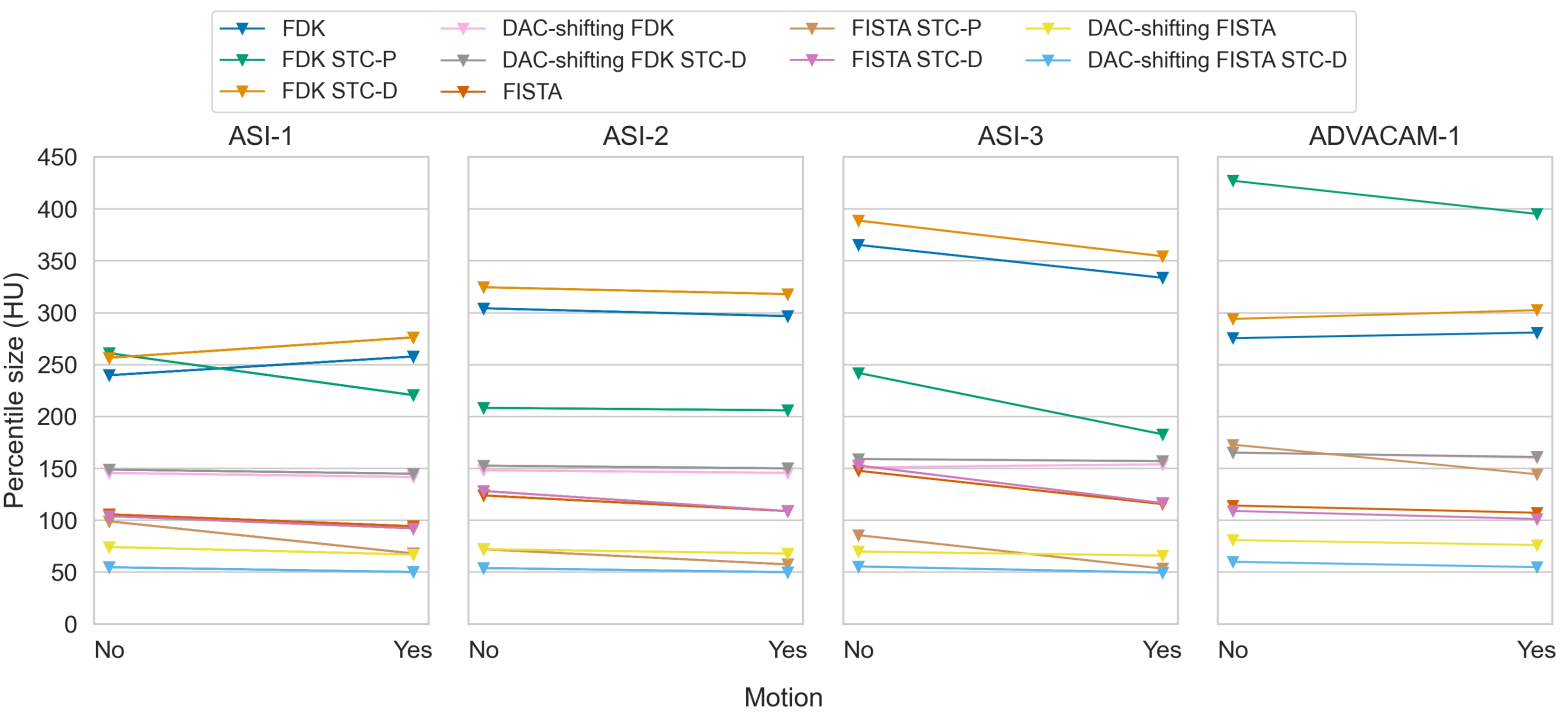
Paired mean of median percentile sizes without and with detector motion for all datasets and all combinations of reconstruction type, DAC-shifting, STC-P, and STC-D. Note that these comparisons are actually comparisons between different acquisitions, as detector motion was an acquisition parameter, not a post-processing method.

The detector motion method effectively translated the ring artefacts caused by pixel response differences into random-seeming noise in the reconstructions (Figure 8). As this method changes the appearance of the image, but not the value range within the materials, the mean percentile sizes were not substantially affected most of the time (Figure 9). The combined effectiveness score for detector motion was limited to −8.7% (IQR −13.0–−1.5%), p<0.001.

### 3.4. Stc-P Beam Hardening and Pixel Response Correction

**Figure 10:**
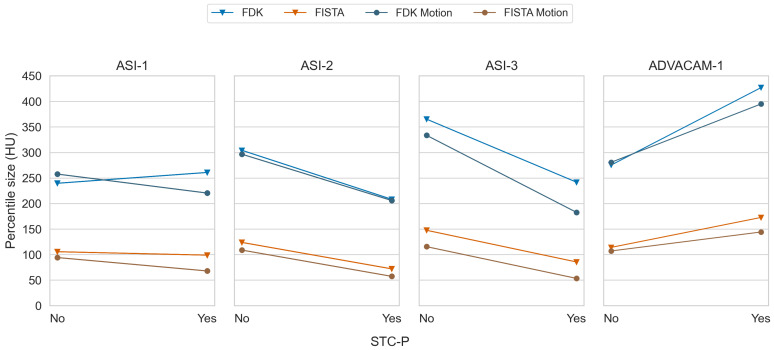
Paired mean of median percentile sizes without and with STC-P for all datasets and all combinations of reconstruction type and detector motion.

**Figure 11:**
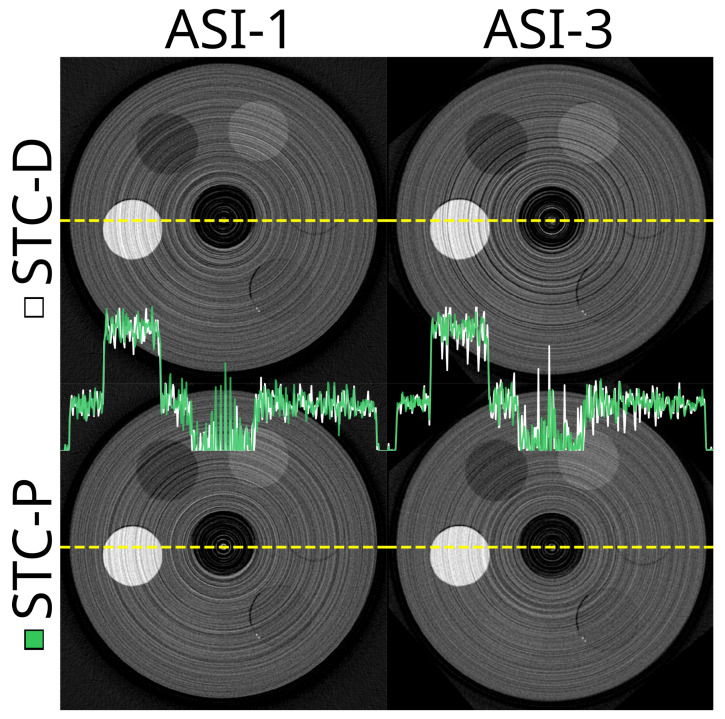
The same slice at 20.0 keV when using the signal-to-thickness calibration with detector-wide data (STC-D) compared to per pixel data (STC-P). The top row is based on ‘ASI-1’ data, and the bottom row is based on ‘ASI-3’ data. The viewing window is set to −1000–1800 HU for all subplots.

We observed substantial differences in the effectiveness of STC-P with the different datasets (Figure 10). The effect of STC-P on two different datasets is visualised in Figure 11. Its corresponding effectiveness scores varied in range from −10.0 to −43.7% for the ASI data, and even a worse image quality for the ADVACAM-1 data (effectiveness score +45.5% (+39.2: +52.3), Table 4). The two datasets that were acquired close to the date of acquiring the STC-D calibration measurements (ASI-2 and ASI-3) seemed to benefit more from STC-D than the other datasets.

### 3.5. DAC-Shifting

**Figure 12:**
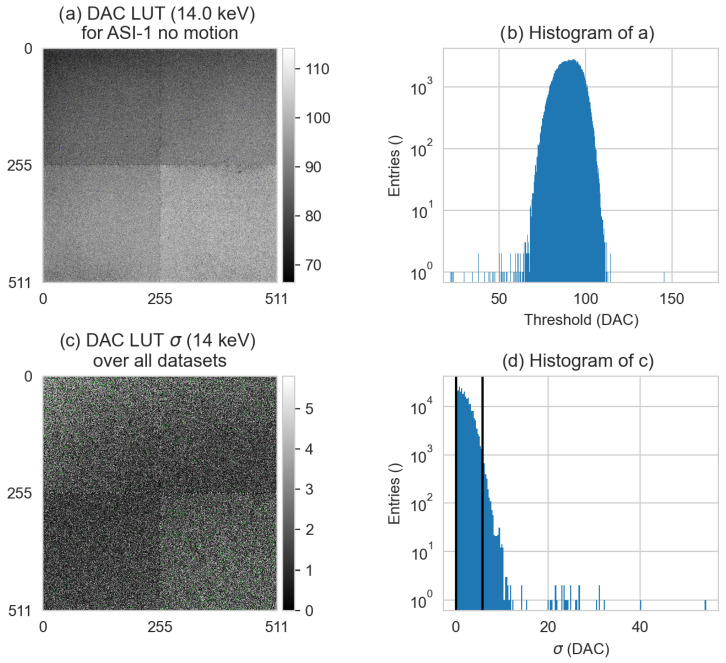
The corrected DAC distributions (LUT) and their standard deviations as a function of the dataset at 14.0 keV. (**a**) The corrected DAC distribution image of the first dataset (#1); the first LUT. (**b**) The corresponding histogram of that image. (**c**) The standard deviation (σ) of all six ASI datasets at 14.0 keV. The window size is set from 0 to 99% (0–5.80 DAC units), and green pixels are above the upper window level. (**d**) The corresponding histogram of (**c**), with the same 99% window size indicated with vertical black lines. The dataset numbers are detailed in Table 2.

The DAC-shifting method focuses on correcting individual pixel responses using a LUT based on the flat-field images of the day. An example of a LUT for a specific threshold and its variation over different datasets can be seen in Figure 12. In this example, the 95% range of the DAC settings for individual pixels that correspond to 14 keV stretches from 73 to 105 DAC units. Its stability over different datasets seems random and the root mean square (RMS) standard deviation of Figure 12c corresponds to 2.33 DAC units, which typically corresponds to 0.47 keV.

**Figure 13:**
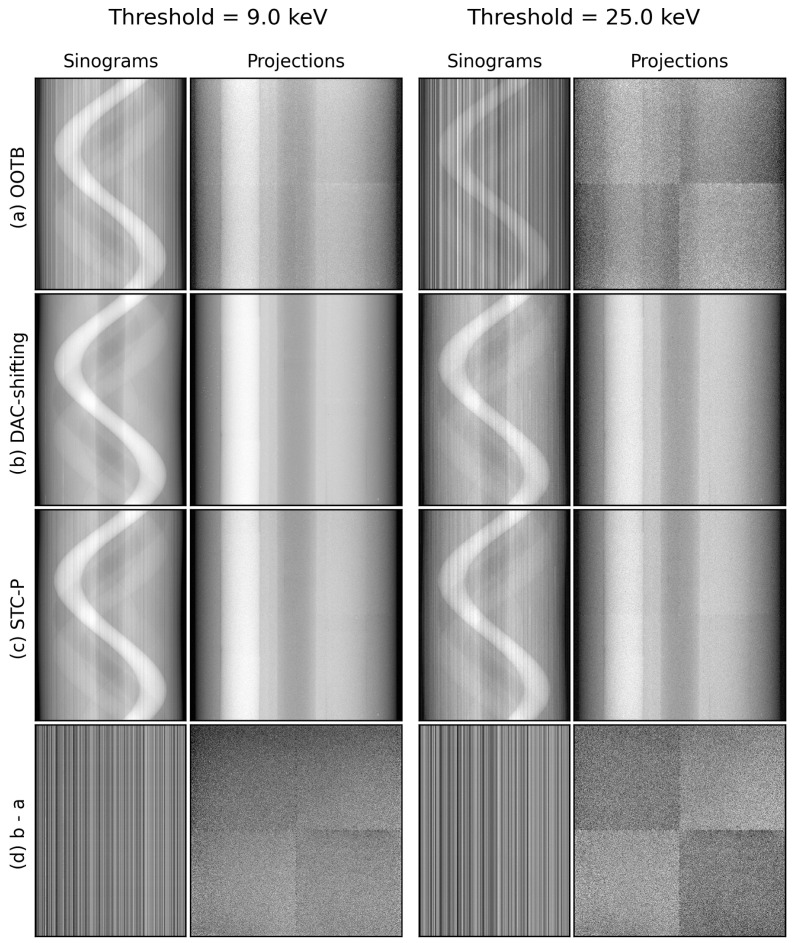
The sinograms and projections of (**a**) OOTB (‘out-of-the-box’), (**b**) DAC-shifted, and (**c**) STC-P data and (**d**) the differences between two of these. The threshold is set to 9.0 or 25.0 keV, respectively, left vs. right. The projections are an arbitrarily selected slice, 88 of 510, from the corresponding sinograms. The viewing windows are all set to 0.1–99.9% of the range per image. In the last row, (**d**), the difference between DAC-shifting (in **b**) and OOTB (in **a**) is shown to illustrate the DAC-shifting correction. The ‘ASI-3’ no-motion dataset was used.

In Figure 13, we illustrated the effect of DAC-shifting on projections and sinograms at two thresholds and compared it to STC-P. Note the significant reduction in vertical stripes in the sinogram images, especially at the higher threshold. In addition, individual chips can be distinguished in the OOTB and STC-P projections, but not in the DAC-shifting projections.

**Figure 14:**
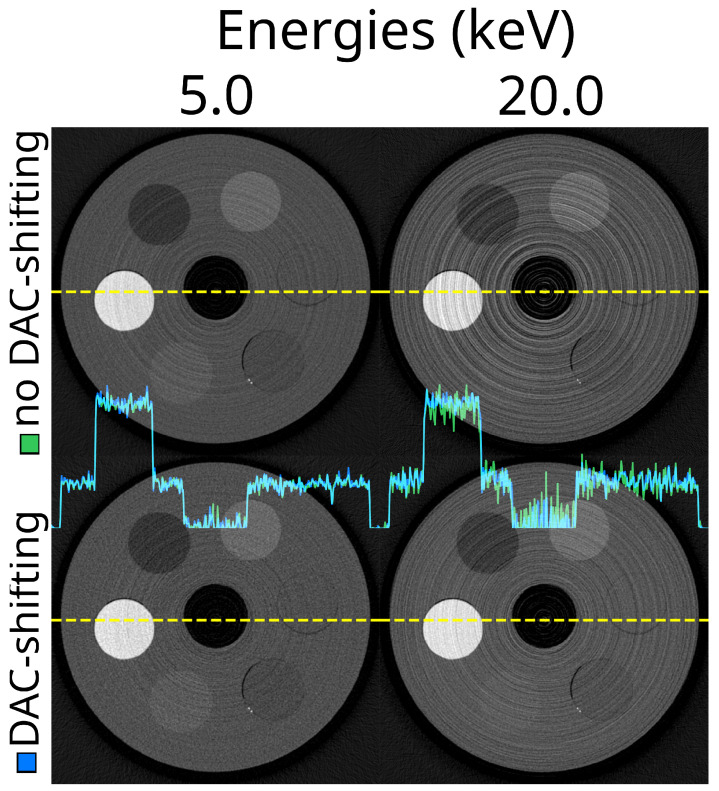
Example reconstructions of different thresholds with and without DAC-shifting. Top row: FDK-reconstructed slice at 5.0 (left) and 20.0 (right) keV using a static detector, STC-D, and no DAC-shifting. The lower row shows the exact same reconstructions, but with DAC-shifting enabled. In the middle of the image, the profile of the yellow dotted lines is green for the top row, while the lower row is blue.

**Figure 15:**
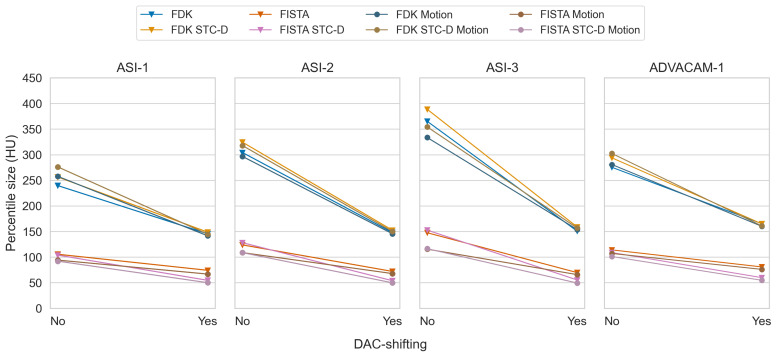
Paired mean of median percentile sizes without and with DAC-shifting for all datasets and all combinations of reconstruction type, detector motion, and STC-D.

With DAC-shifting, ring artefacts were substantially reduced without a degradation of spatial resolution or introduction of obvious artefacts (Figure 14). DAC-shifting also resulted in comparable image quality between different datasets (Figure 15). The absolute mean percentile sizes with DAC-shifting are comparable between the datasets, even when their absolute numbers without DAC-shifting vary (for example, ASI-1 vs. ASI-3). As a result, the effectiveness scores were substantial (mean −47.4%) and varied based on the initial image’s quality (−40.7–−55.6%), p<0.001.

### 3.6. Reconstruction Methods

**Figure 16:**
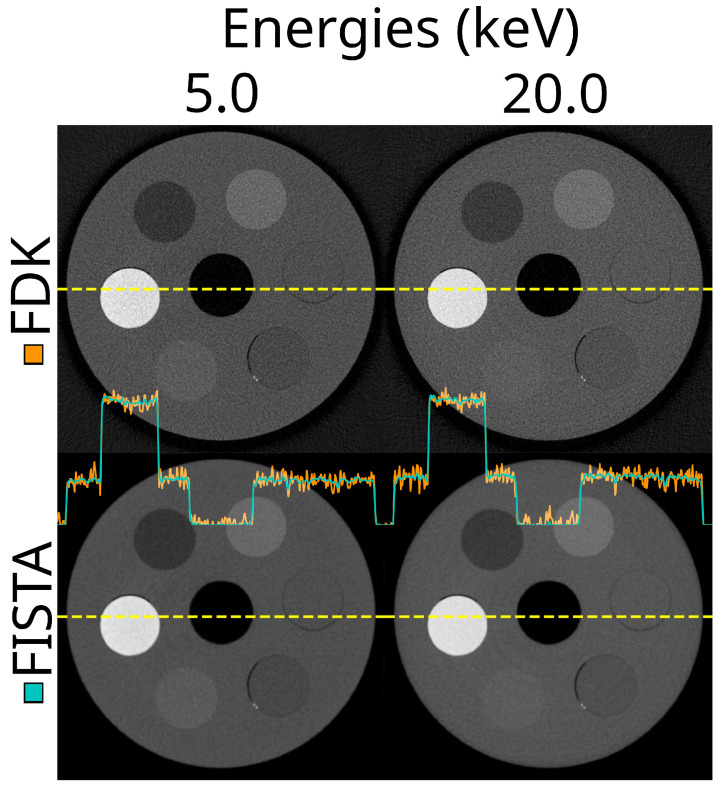
The same reconstructed slice at two energy thresholds (5.0 and 20.0 keV) using the FDK (non-iterative) and FISTA (iterative) reconstruction methods. This is using the ASI detector (ASI-1), STC-D beam hardening correction, detector motion, and DAC-shifting.

So far, we have visualised reconstructions using only the FDK method. A comparison between the iterative reconstruction algorithm, FISTA, and FDK is shown in Figure 16. The FISTA method used in this work was very effective in reducing the noise in the images, with minimal compromise of the image sharpness. The noise reduction clearly translated into reduced mean percentile differences. As a result, the effectiveness score was −61.6% (IQR −66.0–−59.1%), p<0.001.

### 3.7. Optimal Settings

**Figure 17:**
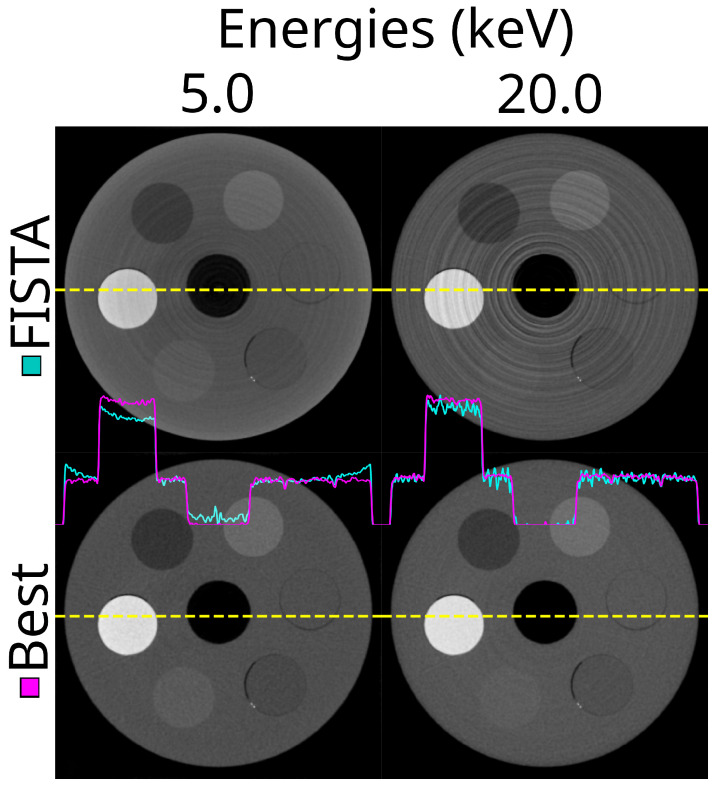
The same reconstructed slice at two energy thresholds (5.0 and 20.0 keV) using the out-of-the-box approach with FISTA, no DAC-shifting, no STC, and no detector motion (top row) and the best combination of all options (lower row). This is using the ASI detector (ASI-1). For the ‘best’ combination, STC-D, detector motion, DAC-shifting, and the FISTA reconstruction method are used.

**Figure 18:**
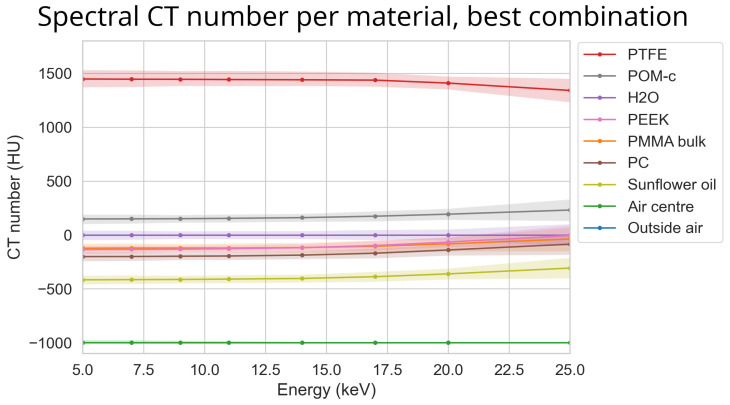
The reconstructed CT number of each material at different energies using the ASI detector for the best parameter combination. Here STC-D, detector motion, DAC-shifting, and the FISTA reconstruction method are used on the ‘ASI-1’ dataset. The corresponding mean percentile size for these data was 49 HU.

The best combination of options was detector motion, STC-D, DAC-shifting, and FISTA reconstruction. Even though the effectiveness score for detector motion was close to zero, it complemented the FISTA reconstruction. This is because detector motion smears the rings into noise, which can effectively be reduced by the FISTA reconstruction. An illustration of the image’s improvement compared to the out-of-the-box setting using FISTA can be seen in Figure 17. The spectral data of the best parameter combination can be seen in Figure 18.

### 3.8. Evaluation of DAC-Shifting Iodine K-Edge Imaging

**Figure 19:**
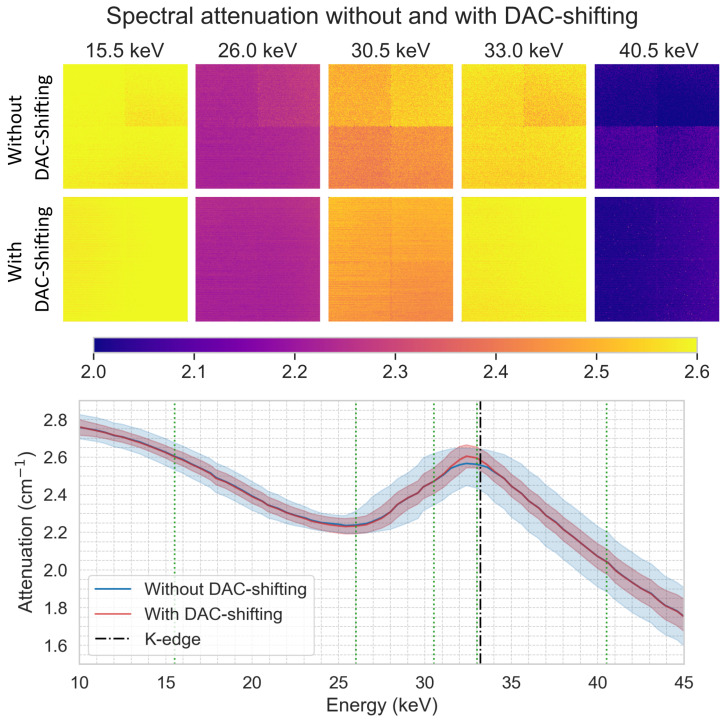
The top row shows a series of attenuation images at various thresholds (15.5, 26.0, 30.5, 33.0, and 40.5 keV), acquired without DAC-shifting. The second row displays images at the same energy level with DAC-shifting. On the bottom row, the plot shows the mean attenuation as function of energy (at 0.5 keV interval) and the 95% data distribution of all chips combined without and with DAC-shifting. The vertical dashed line indicates the K-edge of iodine (33.2 keV) and the green dotted lines indicate the thresholds presented in the top rows.

The capability of the DAC-shifting method to enhance the sharpness of the iodine K-edge using 2D spectral imaging is illustrated in Figure 19. These spectral profiles show that attenuation increases as the energy approaches the K-edge and decreases after passing it. Without DAC-shifting, individual pixel response variations led to a relatively wide 95% data range. In contrast, the DAC-shifting method improved homogeneity between individual chips (see attenuation images) and pixels (see 95% data range), resulting in a sharper attenuation peak near the K-edge.

**Figure 20:**
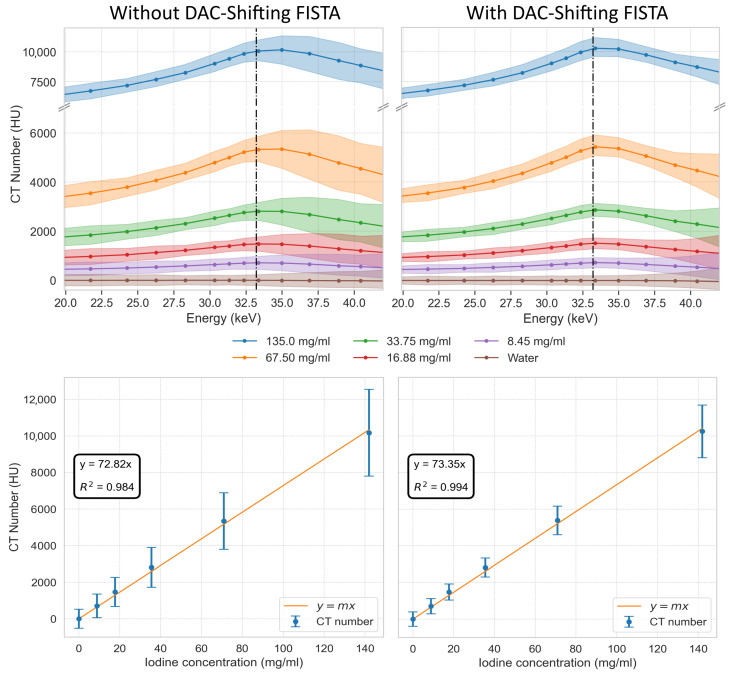
Spectral profiles and concentration calibration of iodine solutions. The top row displays the CT numbers in Hounsfield units (HUs) across a range of energies for iodine concentrations at 135.0, 67.50, 33.75, 16.88, and 8.45 mg/mL, as well as water, without (**top left**) DAC-shifting using FISTA reconstruction and with (**top right**) DAC-shifting using FISTA reconstruction. The vertical dashed lines indicate the K-edge of iodine (33.2 keV). The bottom row illustrates the linear relationship between the varying iodine concentrations at 33.0 keV for FISTA without DAC-shifting (**bottom left**) and FISTA with DAC-shifting (**bottom right**) and their corresponding CT numbers, along with the 95% distribution of the data as vertical error bars.

In 3D spectral iodine imaging, spectral profiles, depicted in CT numbers (HUs) as a function of energy, still show increased CT numbers around the K-edge, but less pronounced than in 2D imaging (Figure 20). Note that the highest concentration in the 3D imaging was half of the concentration in the 2D imaging. The effect of DAC-shifting was again mainly visible in the reduction in the 95% data range, especially at higher thresholds. This resulted in less overlap between the 95% data ranges of the lower iodine concentrations, as can be appreciated from the linear relationships depicted in Figure 20, which is a comparison of R^2^ values, demonstrating the highly significant difference (*p* < 0.001) between the methods without DAC-shifting and with DAC-shifting.

## 4. Discussion

In this paper, we presented a novel post-processing method to deal with individual pixel responses in spectral imaging using PCDs. We have shown how the DAC-shifting method has a positive impact on image quality in relation to existing methods. We have also shown that DAC-shifting is robust against long-term variations in the imaging system, while STC-P did not provide consistent image quality improvements over time.

When comparing the DAC-shifting method with the STC-P method, we can see that, at best, with the most optimal settings, they perform almost comparably. In the ASI datasets with motion, the mean percentile sizes for STC-P with FISTA were 68, 57, and 53 HU for ASI-1, ASI-2, and ASI-3, respectively (Figure 10). For DAC-shifting in combination with STC-D and FISTA reconstruction, the mean percentile sizes were 50, 50, and 49 HU (Figure 15). In general, the results of DAC-shifting were very stable when comparing the effect of the different post-processing methods on the different datasets, even when comparing the two detectors (Figure 15). For STC-P, the changes in mean percentile size were much less stable, and it appears that this only worked for ASI-2 and ASI-3, which were acquired within four days of the STC calibration measurements. These findings exemplify the instability of this imaging system; this has been observed in previous work using a Medipix3-based system [17]. This instability was further illustrated and quantified in the LUT variation example in Figure 12, where the RMS of the standard deviation map was 2.33 DAC units, which corresponds to a 0.47 keV standard deviation in this system. In other words, within the data acquired in a four-week window, individual pixel responses at 14.0 keV can vary between −1.0 and +1.0 keV. Given the random distribution of the variation in the example image, this variation can be attributed to the detector, not the X-ray source.

Even though several papers are available on STC-P, none of them describe the relation between system stability and its effectiveness ([9,10,11,15]). This makes a direct comparison to other papers challenging. The most comparable work in terms of imaging with Medipix3 and at low threshold energies is the work by Ronaldsen et al. using the MARS-CT scanner in 2012 [2]. The spectral profile of sunflower oil obtained in our work (Figure 18) seems directly comparable to their work, with slowly increasing CT numbers above a 15 keV threshold. Unfortunately, they only mention a standard deviation uncertainty of ~10 HU, without explicitly showing the data. They make use of a combined wavelet–Fourier filtering method for ring artefact correction [18], but the effect of this is not separately illustrated. They also imaged iodine, at a concentration of 0.01 mol/L, which is lower than our lowest concentration (0.033 mol/L). However, they only measured thresholds of 30.9 and 36.2 keV around the K-edge, at which the K-edge was not directly visualised.

He et al. performed a more comparable K-edge imaging of iodine, both in 2D and 3D [6]. They showed similar graphs regarding the K-edge in terms of its attenuation at different energies (Figure 19). In their and our work, the ability to precisely resolve the K-edge is limited by the effective spectral resolution of the detector used. The spectral profile appears to be between the theoretical mass attenuation examples at 1.0 and 3.0 keV $\sigma_{E}$ (energy resolutions) provided by Ge et al. [6]. In their example, they show that a limited energy resolution convoluted with the sharp K-edge, which shifts the attenuation peak to a slightly higher energy.

When calibrating the Medipix3 detector, an equalisation procedure minimises the variation in pixel response at a specific threshold. In our setup, we focused our research on low keV thresholds. The Advacam detector was equalised at approximately 8.0 keV, whereas the ASI detector was configured using the noise edge, which is approximately 4.0 keV. Due to threshold dispersion, where the pixel responses diverge as the threshold is increased, the DAC-shifting improvement was more significant at higher thresholds, achieving comparable image quality between 5.0 and 20.0 keV (Figure 17). Alternative methods like loading specific equalisation maps for each threshold used could help increase the image quality of the raw data. Currently, this step would increase the acquisition time substantially, as the loading of the equalisation maps takes time.

Due to the instability of our imaging system, the STC-P method did not provide consistent image quality improvement. To make use of the advantage of STC in correcting for beam hardening, we introduced the STC-D method. To the best of our knowledge, this approach has not been used before. In our setting, the advantage was that STC-D could be combined with the DAC-shifting method. The method is simple, reliable, and fast because it can be applied to the entire stack of projection data at once and is less sensitive to variations over time. Of course, the effectiveness of the STC-D beam hardening correction is dependent on the materials used for calibration, PMMA in our case, and the sample that is imaged. For the phantom with plastic inserts it worked well, but for the iodine acquisitions it was less effective; this was at least partly due to the minimal beam hardening at higher energies.

In recent years, there has been a focus on improving spectral image quality by utilising dedicated spectral reconstruction methods with material decomposition [19,20,21] and deep learning-based reconstruction for noise reduction [22,23], or combinations thereof [24]. While these novel methods all seem very promising, they could still take advantage of the presented DAC-shifting method. This method corrects the projection data, keeping the spectral integrity without changing the units, and homogenises data between the different chips and pixels. In this way, the input data used for reconstruction are improved, providing improvements for downstream steps like material decomposition. The challenge with our method is that it adds measurement and processing time. It took between 10 and 12 min to apply DAC-shifting on a modern 64 core computer (AMD Ryzen Threadripper 3990X) utilising Python and parallel processing on 60 of the cores for most of the steps. To speed this up, there are many options, one of which is a neural network for the DAC-shifting method to improve its processing speed. The neural network then needs to perform the spectral fitting and determine the corrections for the projection data. This can most likely be achieved using a physics-informed neural network [25], with which we can use the raw and DAC-shifted images for supervised learning. The speed-up then comes from the highly parallelised GPU processing of the data.

In this work, we have only used Medipix3 detectors with a silicon sensor, but the method can in principle be used with any type of spectral photon-counting detector or sensor material. Sensor materials with K-edges in the region of scanning may introduce complications; this was not investigated in this work. The minimum requirement is that multiple spectral thresholds need to be acquired, including flat-field images. Example detectors could be the Dectris PILATUS series [26] and also the Timepix family used in frame-based readouts or time-over-threshold modes [27].

The presented DAC-shifting method has some limitations. First of all, the method is based on performing cubic b-spline fits through the spectral data, which means that it can only be used on spectral data and not individual single-threshold data. The cubic b-spline approach does not utilise any knowledge about the underlying physics. Ideally, we would fit a function which is related to the underlying physics, with a data-based uncertainty estimation to constrain the fits. Secondly, at the edges of the spectral data, the lowest and highest threshold, approximately half of the pixels were corrected using the extrapolation of the data, which is less accurate than interpolation due to the cubic b-spline approach. This effect was visible in Figure 18, where the 95% data range increased at the highest threshold plotted. Note that in our work we already used one extra threshold acquisition (Table 1 #5, threshold 0 = 25.0 keV; threshold 1 = 30.0 keV) to mitigate this issue. These data were only used to guide the spline fitting, but the DAC-shifted reconstructions at these thresholds did not result in improved image quality. This is due to the extrapolation. Thirdly, since each counter corresponds to different transistors, and therefore each counter has individual response patterns, the DAC-shifting method was applied per counter. This means that the approach is currently not applicable to PCDs that have many more counters per pixel, where the entire spectral profile can be acquired at once. Further research is needed to address this methods’ use in these detectors; a first approach was taken in [17]. Finally, we reconstructed our data as spectral thresholds, not spectral bands (energy bins), which are often presented in other papers. However, the DAC-shifting method is applicable in both cases. When spectral bands are needed, the DAC-shifting method can be applied first to the threshold data, and subsequently spectral bands can be calculated by subtracting the two thresholds.

## 5. Conclusions

Within the context of improving the reconstruction quality of our system, we have presented a novel post-processing method that minimises individual pixel responses in spectral photon-counting cone-beam CT. This method was shown to be consistently effective in correcting the sinogram data, which resulted in reduced ring artefacts and improved image quality. On average, DAC-shifting resulted in a 47% reduction in CT number variation within homogeneous materials. This was furthermore illustrated with improved iodine concentration measurements. This method does not introduce artefacts, maintains the scale and units of the data, and was shown to provide consistent image quality, even with different-quality input data.

## Figures and Tables

**Table 1:** An overview of the thresholds and exposure times used in the acquisitions.

Acquisition Number	Threshold 0 (keV)	Threshold 1 (keV)	Exposure Time (s)
1	5.0	7.0	2.08
2	9.0	11.0	2.88
3	14.0	17.0	4.48
4	20.0	25.0	9.60
5	25.0	30.0	9.60

**Table 2:** Overview of the measurements performed. On 20 December 2023 measurements were taken for the STC calibration of both detectors.

Measurement Number	Date	Manufacturer	Motion	Days from STC Calibration	Dataset Name
1	29 November 2023	ASI	No	−22	ASI-1
2			Yes		
3	30 November 2023	ADVACAM	No	−21	ADVACAM-1
4			Yes		
5	19 December 2023	ASI	No	−1	ASI-2
6			Yes		
7	23 December 2023	ASI	Yes	+3	ASI-3
8	24 December 2023		No	+4	

**Table 3:** Overview of the acquisition numbers, thresholds, and exposure times used in the 3D spectral CT acquisition of different iodine concentrations.

Acquisition Number	Threshold 0 (keV)	Threshold 1 (keV)	Exposure Time (s)
1	19.5	21.5	1.10
2	24.0	26.0	1.66
3	28.0	30.0	3.04
4	31.0	32.0	4.00
5	33.0	35.0	5.10
6	35.0	37.0	6.04
7	37.0	39.0	7.44
8	40.5	42.5	10.0
9	42.5	44.5	10.0

**Table 4:** This table presents a comparison of the effectiveness of different methods at improving image quality across various datasets. A combined analysis was performed using all the listed datasets, in addition to individual dataset analyses. The results are presented as effectiveness scores, which are percentage changes in image quality. The scores are given as means and interquartile ranges (IQRs). *p*-values are also provided to indicate the statistical significance of the findings. Significant results are highlighted in bold. The effectiveness scores allow for a quantitative comparison of the different methods across the various datasets, both individually and collectively.

Datasets	STC-D	Detector Motion	STC-P	DAC-Shifting	Reconstruction Type
ASI-1	−4.6 (−7.9: +3.4) *p* = 0.97	−7.8 (−11.3: −2.7) *p* = 0.22	−10.0 (−17.8: −2.5) *p* = 0.41	**−40.7 (−46.1: −36.9)** ***p* = 0.001**	**−60.7 (−64.9: −56.8)** ***p* < 0.001**
ASI-2	−3.6 (−6.3: +4.3) *p* = 0.67	**−7.0 (−11.0: −1.8)** ***p* < 0.001**	**−37.8 (−43.2: −31.3)** ***p* < 0.001**	**−50.0 (−53.3: −48.6)** ***p* < 0.001**	**−62.3 (−65.7: −59.6)** ***p* < 0.001**
ASI-3	−2.7 (−4.5: +5.6) *p* = 0.48	**−14.1 (−23.3: −6.4)** ***p* < 0.001**	**−43.7 (−47.4: −40.0)** ***p* < 0.001**	**−55.6 (−58.8: −53.6)** ***p* < 0.001**	**−63.3 (−66.7: −59.9)** ***p* < 0.001**
ADVACAM-1	−6.2 (−10.7: +2.1) *p* < 0.78	−5.3 (−7.4: −2.6) *p* = 0.08	+45.5 (+39.2: +52.3) *p* = 0.04	**−40.3 (−45.2: −37.3)** ***p* = 0.002**	**−60.6 (−63.7: −58.8)** ***p* < 0.001**
Combined	−4.2 (−7.4: +3.6) *p* = 0.83	**−8.7 (−13.0: −1.5)** ***p* < 0.001**	**−13.7 (−16.6: −10.4)** ***p* < 0.001**	**−47.4 (−51.1: −46.3)** ***p* < 0.001**	**−61.6 (−66.0: −59.1)** ***p* < 0.001**

## Data Availability

Imaging data that were used in this study can be shared upon request.
